# Long‐read sequencing of recurrent *FGF12* duplications in epilepsy: Insights into structural mechanisms and aberrant isoforms

**DOI:** 10.1111/epi.18609

**Published:** 2025-08-21

**Authors:** Jade Fauqueux, Laurence Chaton, Pierre Cleuziou, Anne‐Sophie Diependaële, Nathalie Bach, Nicolas Gruchy, Marion Gerard, Jean‐Pascal Meneboo, Céline Villenet, Martin Figeac, Emilie Ait‐Yahya, Caroline Thuillier, Elise Boudry, Adeline Trauffler, Sylvie Nguyen‐The‐Tich, Simon Boussion, Roseline Caumes, Jamal Ghoumid, Thomas Smol

**Affiliations:** ^1^ Univ. Lille, Unité de Recherche ULR7364 RADEME ‐ Maladies RAres du DÉveloppement Fédération Hospitalo‐Universitaire FHU‐G4 Génomique Lille France; ^2^ Centre Hospitalier Universitaire de Lille Service de Neurophysiologie Clinique Lille France; ^3^ Centre Hospitalier Universitaire de Lille Clinique de Pédiatrie, Service de Neuropédiatrie Lille France; ^4^ Centre Hospitalier Universitaire de Caen Service de Neurologie Unité Explorations Fonctionnelles Neurologiques Caen France; ^5^ Centre Hospitalier Universitaire de Caen Service de Pédiatrie Caen France; ^6^ Univ. Caen Normandie, Unité de Recherche UR7450 BioTARGen, Fédération Hospitalo‐Universitaire FHU‐G4 Génomique Centre Hospitalier Universitaire de Caen, Service de Génétique Caen France; ^7^ Univ. Lille, Centre National de la Recherche Scientifique CNRS, Inserm, Centre Hospitalier Universitaire de Lille Institut Pasteur de Lille, Plateformes Lilloises en Biologie & Santé Lille France; ^8^ Centre Hospitalier Universitaire de Lille, Cellule de Bioinformatique Plateau Commun de Séquençage Lille France; ^9^ Centre Hospitalier Universitaire de Lille Institut de Génétique Médicale Lille France; ^10^ Centre Hospitalier Universitaire de Lille Clinique de Génétique Lille France

**Keywords:** FGF12, intellectual disability, long‐read sequencing cDNA analysis

## Abstract

**Objective:**

Fibroblast growth factor 12 (*FGF12*), a member of the fibroblast homologous factor family, plays a key role in the modulation of voltage‐gated sodium (Nav) channels. Pathogenic variants in the *FGF12* gene leading to a gain‐of‐function mechanism and partial duplication encompassing the *FGF12* gene leading to a loss‐of‐function mechanism are associated with developmental and epileptic encephalopathy (DEE), characterized by developmental delay, intellectual disability, ataxia, and drug‐resistant epilepsy. We report two patients with DEE harboring de novo recurrent intragenic duplications of *FGF12* identified by long‐read sequencing (LRS).

**Methods:**

We applied LRS to the DNA and cDNA of patients with *FGF12* duplication to fully characterize the DNA's structural organization and its transcriptional consequences. Additionally, we reanalyzed electroencephalographic (EEG) data from patients at different timepoints to identify phenotypical specificities and refine the electroclinical spectrum.

**Results:**

These duplications, spanning approximately 536 kbp, were mediated by nonallelic homologous recombination between L1PA2 elements (LINE‐1 Primate‐specific subfamily A, number 2). cDNA analysis revealed aberrant transcripts, one predicted to encode an elongated FGF12 protein and another leading to premature termination. Both patients shared overlapping clinical features, including postepilepsy onset regression, global developmental delay, and ataxia. EEG studies revealed a marked early encephalopathic pattern with disorganized and high‐voltage slow background activity with multifocal spikes at onset evolving later into subcontinuous generalized spike and wave activation.

**Significance:**

Our findings are consistent with previous reports linking structural variants to functional disruption, suggesting impaired Nav channel activity due to a shift in inactivation to hyperpolarized potential, leading to a loss‐of‐function effect. These findings underscore the utility of LRS for DNA and cDNA analysis in resolving structural variants and expanding the electroclinical spectrum of patients with *FGF12* duplications.

**Key points**

- LRS identified aberrant *FGF12* transcripts, including an elongated protein and a truncated variant.
- *FGF12* duplications in DEE impair Nav channel function, leading to a loss‐of‐function effect and epilepsy‐related regression.
- EEG analysis showed an early encephalopathic pattern, with high‐voltage slow activity and multifocal spikes evolving into generalized spike–waves.

## 1 INTRODUCTION

Fibroblast growth factor 12 (FGF12), encoded by the *FGF12* gene and previously designated FHF1, is a member of the fibroblast growth homologous factor (FHF) family, which includes *FHF1* (*FGF12*), *FHF2* (*FGF13*), *FHF3* (*FGF11*), and *FHF4* (*FGF14*).^1^ *FGF12* is predominantly expressed in the developing and mature nervous system, cardiac and skeletal muscle tissues, and olfactory epithelium.^2^ The *FGF12* transcripts produce two major transcripts. The longer transcript, designated A, produces a protein with an N‐terminal nuclear localization‐like sequence (NLS) that allows its nuclear localization. In contrast, the shorter transcript, designated B, produces a protein without the NLS sequence, resulting in its cytoplasmic localization.^1^, ^3^ FGF12 interacts with voltage‐gated sodium (Nav) channels, including Nav1.2 (SCN2A), Nav1.5 (SCN5A), and Nav1.6 (SCN6A), and modulates the fast inactivation kinetics of these channels.^2^, ^4^

Recurrent pathogenic heterozygous missense variants in *FGF12* have been associated with autosomal dominant developmental and epileptic encephalopathies (Mendelian Inheritance in Man #617166), and have been reported in more than 20 publications.^5^, ^6^, ^7^, ^8^, ^9^ The associated phenotype includes developmental delay, intellectual disability, axial hypotonia, ataxia, and early onset drug‐resistant epilepsy.^10^ Electrophysiological studies have demonstrated a gain‐of‐function effect of these recurrent variants, which causes depolarizing shifts in the voltage‐dependent fast inactivation of Nav1.6.^5^, ^11^ In addition to single nucleotide variants, partial duplications of the *FGF12* gene with a similar phenotype have been reported in six patients.^12^, ^13^, ^14^ The duplication is expected to produce a longer transcript associated with a loss‐of‐function mechanism.^12^, ^15^

We report two additional patients carrying a recurrent partial duplication of *FGF12*. Using DNA long‐read sequencing (LRS), we fully resolved the underlying structural variant without prior assumptions, demonstrating that it corresponds to a direct tandem duplication. Additionally, we examined aberrant *FGF12*‐bearing transcripts using full‐length cDNA LRS. We also provide a detailed description of the clinical and electroclinical phenotypes of the disease. This study highlights the central role of LRS in elucidating structural variants and offers new insights into their functional consequences.

## 2 MATERIALS AND METHODS

### 2.1 Ethics statement

Clinical details were collected, and informed consent was obtained for genetic studies. Blood samples were collected from the affected individuals and their parents, after informed consent was obtained. Analyses were performed on a diagnostic basis in accordance with the bioethical rules of French law. This study was approved by the Comité de Protection des Personnes ethics committee (reference no. 2023‐A00473‐42). Informed written consent was obtained from all participants or their legal guardians.

### 2.2 Cell culture

Human lymphoblastoid cell lines (LCLs) were established by Epstein–Barr virus immortalization of the subject's blood lymphocytes and maintained in RPMI 1640 medium (Thermo Fisher Scientific) supplemented with 15% fetal bovine serum (Thermo Fisher Scientific) and 1% penicillin–streptomycin (Life Technologies).

### 2.3 DNA and RNA extraction

DNA was extracted from patient lymphocytes using the QIAamp DNA Blood Kit (Qiagen) according to the manufacturer's instructions. RNA was extracted from the LCL of Patient 1 using the PureLink RNA Minikit (Invitrogen).

### 2.4 Array comparative genomic hybridization

Copy number variations were detected by comparative genomic hybridization (CGH) array experiments using a 60‐K oligonucleotide microarray (SurePrint G3 Human CGH Microarray Kit, 8 × 60 K, Agilent Technologies). Data were analyzed using CGH‐Analytics software v2.7 and the ADM2 algorithm.

### 2.5 Nanopore LRS

DNA libraries were prepared using the Ligation Sequencing Kit v14 (Oxford Nanopore Technologies) and subsequently sequenced following the standard protocol on a GridION sequencer (Oxford Nanopore Technologies). After sequencing, base calling was performed using the Guppy base caller (v6.4.6) in high‐accuracy (HAC) mode. Reads were mapped to the human reference genome (GRCh38) using MiniMap2 (v2.24) and NGMLR (v0.2.7).^16^, ^17^ Structural variations were detected using Sniffles2 (v2.0.7) and CuteSV (v2.1.1). Sniffles2 calling was tested with specific parameters to enhance detection of complex variants: “‐‐long‐dup‐length 5 000 000” and “‐‐minsupport 3” and CuteSV with “‐‐max_size −1” and “‐‐min_support 3”. Visual analysis of structural variations in target regions were conducted with the Integrative Genomics Viewer (v2.18).^18^, ^19^

cDNA synthesis was then performed using the SuperScript III First‐Strand Synthesis System Kit (Thermo Fisher Scientific), followed by real‐time polymerase chain reaction (RT‐PCR) using the NEBNext High‐Fidelity Polymerase (New England Biolabs). Several primers were designed to amplify and analyze both wild‐type (WT) and aberrant transcripts based on the proposed hypothesis. The goal was to amplify the junction of the tandem duplication in *FGF12* transcripts. For aberrant transcripts, primers targeting exons 4 to 3 were used, whereas for WT transcripts, primers targeting exons 3 to 6 were used; these primers also amplified aberrant transcripts (primers are available upon request; Figure S1). PCR amplified products were prepared as nanopore libraries and loaded onto a PromethION flow cell. Base calling was performed using the Guppy base caller (v6.4.6) in HAC mode. The resulting reads were aligned to the human reference genome GRCh38 using Minimap2 with splice‐aware alignment settings to accommodate transcriptomic data. All chimeric alignments were mapped to the genome using the BLAST tool to determine their genomic origin, allowing us to distinguish between WT and aberrant transcripts.^20^

### 2.6 Breakpoint analysis

Sequencing primers were designed to amplify the specific breakpoints in the two patients. An initial PCR of approximately 6000 bp was performed using Roche Expand Long Template Polymerase (Roche). This was followed by nested PCR using NEBNext High‐Fidelity 2X Polymerase (New England Biolabs). PCR products were extracted from a 2% agarose gel, purified using a PCR clean‐up kit (Macherey‐Nagel), and sequenced by the Sanger method (primers are available upon request).

## 3 RESULTS

### 3.1 Clinical description and EEG features of Patient 1

**FIGURE 1:**
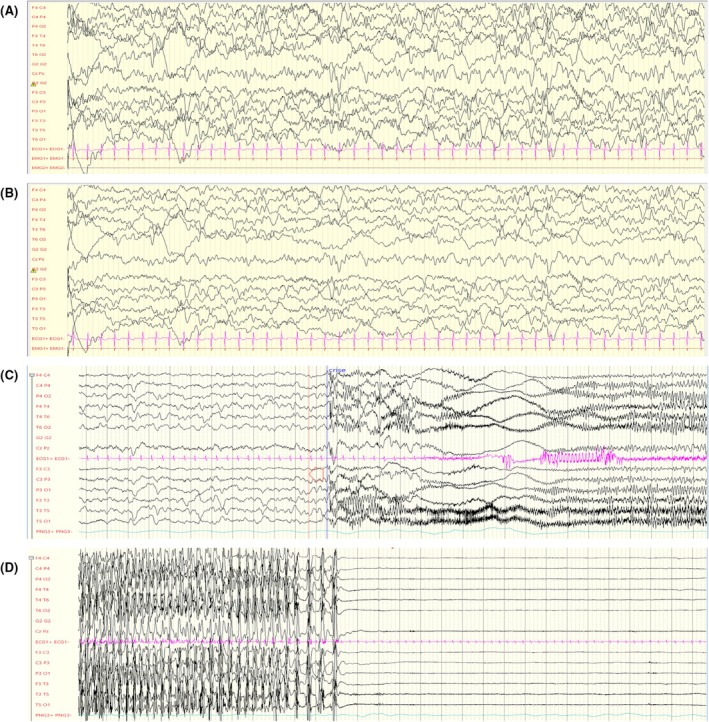
Patient 1 electroencephalographic (EEG) background at onset of epilepsy (acute phase 1) at 18 months in wakefulness (A) and sleep (B) and during seizures (C, D). (A) In wakefulness, abnormally high‐voltage slow disorganized activity between 200 and 400 μV, with rare multifocal spikes. Filter settings: .53–70 Hz, 50‐Hz notch filter; sensitivity: 20 μV/mm. (B) In sleep, with reduced delta activity at 1.5 Hz, and richer in theta at 4–5 Hz; sleep spindles were found bilaterally. Filter settings: .53–70 Hz, 50‐Hz notch filter; sensitivity: 15 μV/mm. (C, D) Bilateral tonic–clonic seizures followed by a suppressed EEG for 1 min after. Filter settings: .53–70 Hz, 50‐Hz notch filter; sensitivity: 25 μV/mm.

Patient 1 was born to healthy unrelated parents after an uncomplicated pregnancy. Early psychomotor development was normal (walking at 14 months, first words at 12–13 months, good social interactions). No dysmorphic features could be seen. Epilepsy began abruptly at 18 months of age, with recurrent afebrile generalized motor status epilepticus (three status epilepticus incidents in 1 month) and frontotemporal seizures with rapid bilateralization. First electroencephalography (EEG), performed in the context of multiple daily seizures, showed disorganized, slowed (3.5 Hz on average, delta predominant 1.5 Hz intertwined with theta 4–5 Hz), and atypical very high‐voltage background activity (150–400 μV) with no anteroposterior gradient in wakefulness and rare focal spikes often diffuse over one hemisphere, either right or left (Figure 1A). Delta activity decreased in sleep, whereas theta activity increased, and bilateral sleep spindles were present (Figure 1B). EEG monitoring recorded bilateral tonic–clonic seizures, lasting 1–1.5 min, with a myoclonic jerk, tonic phase, and bilateral clonic phase (Figure 1C,D) and seizures with frontotemporal onset before bilateralization. A few weeks after the onset of epilepsy, the patient showed severe psychomotor regression, including complete loss of oral language, severe relational disorder (lack of eye contact, no smiling, laughing, or crying), pyramidal syndrome, loss of gait, and ataxia. Initial magnetic resonance imaging (MRI), cerebrospinal fluid analysis, and immunologic and metabolic tests were normal. The patient had no extraneurological involvement and a normal cardiac evaluation. Given the severity of the disease, he was treated with immunoadsorption at 20 months of age on the assumption of seronegative encephalitis. Because it resulted in a partial clinical improvement (regaining the ability to walk), this was followed with rituximab infusions for 18 months.

**FIGURE 2:**
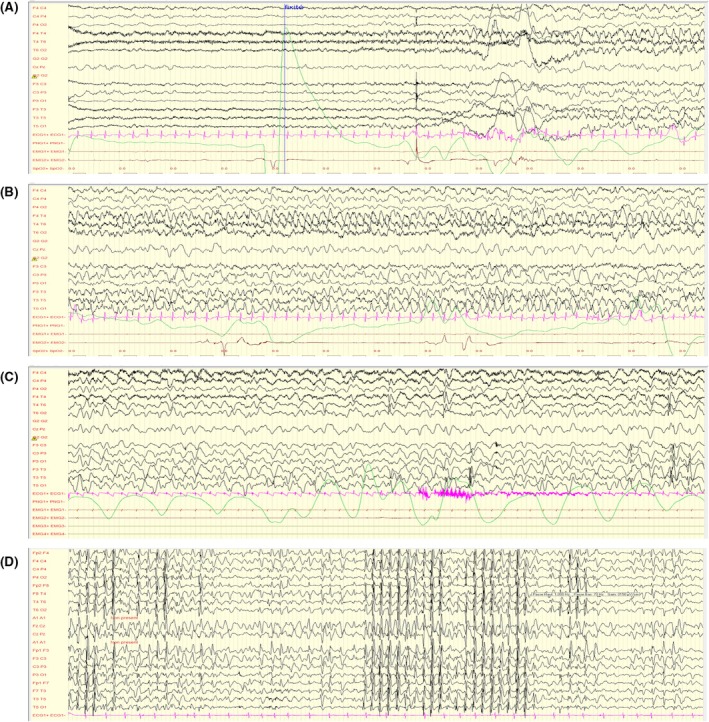
Patient 1 electroencephalographic (EEG) background at age 3 years during seizures (“honeymoon” phase 2 with intermittent seizures; A, B) and 1 month later (C), and at last follow‐up at 6 years old (phase 3 aggravation; D). (A, B) Bilateral tonic–clonic seizures followed by a suppressed EEG for 1 min after. Filter settings: .53–70 Hz, 50‐Hz notch filter; sensitivity: 25 μV/mm. (C) Slow hypersynchrony on awakening lasting several minutes, generalized delta rhythmic, with altered contact during this period on several successive EEGs and an increase in diffuse triphasic waves. Filter settings: .53–70 Hz, 50‐Hz notch filter; sensitivity: 10 μV/mm. (D) Background of diffuse encephalopathy rich in bi‐ and triphasic delta activity, with isolated generalized spikes or bursts of spikes‐waves at 2.5 Hz, with atypical absence and concomitant myoclonus. Filter settings: .53–70 Hz, 50‐Hz notch filter; sensitivity: 10 μV/mm.

After the initial acute phase, epilepsy evolved with monthly bursts of bilateral tonic–clonic, tonic, tonic with focalization, and multiple types of complex focal seizures lasting 3–5 days. Figure 2A,B presents a 1‐min focal seizure with right temporal onset registered at 3 years old. EEGs showed prolonged slow hypersynchrony on awakening lasting several minutes, with generalized delta rhythm and altered contact (Figure 2C), an increase in diffuse triphasic waves during wakefulness, and more frequent multifocal spikes. Of the 29 EEGs performed over 5 years, only one showed normal voltage, with subnormal background activity and rare paroxysmal activity during sleep, recorded after a 2‐month seizure‐free period. This transient improvement was not related to any therapeutic modification. Evolution of brain MRI revealed mild cerebral and cerebellar atrophy (Figure S2). A positron emission tomography brain scan showed marked, symmetrical hypometabolism in the frontal, parietal, and temporal associative areas.

At the age of 5 years, myoclonic and atypical absence seizures appeared, along with a substantial increase in subcontinuous generalized spikes and waves on the EEG. The frequency of tonic and bilateral tonic–clonic seizures increased, occurring several times per day. Background activity was characterized by isolated generalized spikes or bursts of spike and waves at 2.5 Hz, along with atypical absence seizures and concomitant myoclonus (Figure 2D), intertwined with bi‐ and triphasic delta activity. His neurological condition deteriorated significantly, with complete loss of motor skills, lack of feeding autonomy, and a drastic reduction in daily wake time, with no responsiveness to stimulation.

Since epilepsy started, several antiepileptic therapies were tried. Ketogenic diet, vitamin B6, vitamin B8, and cannabidiol (10 mg/kg/day) were ineffective. Levetiracetam (55 mg/kg/day), sodium valproate (30 mg/kg/day), lamotrigine (poorly tolerated at 1 mg/kg/day), clonazepam (.15 mg/kg/day), clobazam (.15 mg/kg/day), topiramate (poorly tolerated at 2 mg/kg/day), and phenobarbital (5 mg/kg/day) showed transient and partial efficacy. Sodium channel blockers were introduced after genetic diagnosis at the age of 5 years 3 months. Carbamazepine (20 mg/kg/day) temporarily reduced seizure activity, without improvement of background abnormalities on EEG, and lost efficacy after a few months. Phenytoin successfully treated bilateral tonic–clonic status epilepticus, but long‐term use resulted in increased ataxia and worsening myoclonus. At the last follow‐up (6.5 years old), the patient was treated with phenobarbital and felbamate (Figure S3). He remained bedridden, poorly reactive, and experienced tonic and bilateral tonic–clonic seizures several times per day.

### 3.2 Clinical description and EEG features of Patient 2

**FIGURE 3:**
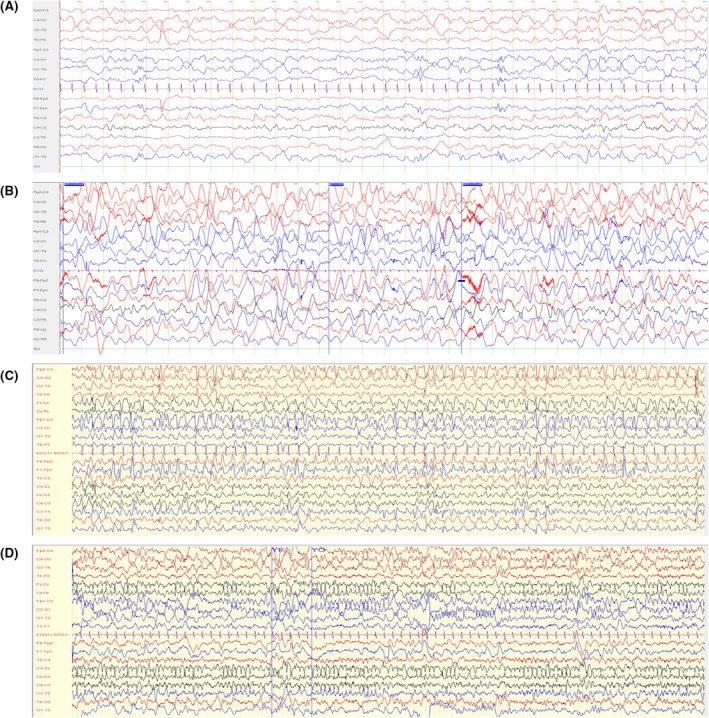
Electroencephalographic (EEG) background of Patient 2. (A) Acute phase 1 at 6 months old during sleep, showing slow waves and spikes in left temporal and occipital regions. Filter settings: .53–70 Hz, 50‐Hz notch filter; sensitivity: 20 μV/mm. (B) At 4 years old, showing slow disorganized background with high‐voltage delta waves. Filter settings: .53–70 Hz, 50‐Hz notch filter; sensitivity: 15 μV/mm. (C) At 6 years old, showing slow disorganized and high‐voltage background with bi‐ and triphasic waves and continuous spikes‐waves in the frontal regions. Filter settings: .53–70 Hz, 50‐Hz notch filter; sensitivity: 20 μV/mm. (D) At 6 years old, showing slow disorganized background with sharp theta rhythmic discharge on central vertex regions. Filter settings: .53–70 Hz, 50‐Hz notch filter; sensitivity: 10 μV/mm.

Patient 2 is the third child of a nonconsanguineous couple with no family history of epilepsy. He was born eutrophic at full term after an uncomplicated pregnancy. Epilepsy began at 5 months of age, with generalized epileptic seizures characterized by hypotonia and nonreactivity lasting approximately 2 min, followed by normal recovery. He rapidly presented recurrent seizures with head deviation and clonic movements of the upper limbs. Initial EEG showed a moderately slowed baseline rhythm, along with slow waves and spikes in the temporal regions during sleep (Figure 3A). Despite the beginning of epilepsy, he reached early developmental motor milestones (sitting at 9 months, walking at 14 months). Language regression was noted at 15 months in the context of serous otitis, followed by an absence of further language development and increasing social withdrawal. A diagnosis of severe autism spectrum disorder was confirmed at the age of 4 years. The patient achieved independent walking, with no signs of pyramidal syndrome or ataxia. He is well integrated into a specialized medical–educational facility. Brain MRI performed at 6 months and at 2 years of age was normal.

He had 1–2 seizures per month during his first years of life. Administered antiepileptic treatments were poorly effective (sodium valproate, levetiracetam, oxcarbazepine, lamotrigine, and topiramate). At approximately 3 years old, EEG revealed global slowing with abnormally high‐voltage and disorganized delta activity (Figure 3B).

At 6 years old, he experienced a significant increase in seizure frequency, consisting primarily of bilateral tonic–clonic episodes that could be prolonged, along with multiple types of focal seizures. Background activity on EEG deteriorated and showed a predominant delta encephalopathy pattern, with frequent bi‐ and triphasic delta waves in addition to bilateral frontal spike–wave discharges (Figure 3C). Further treatment modifications did not provide significant improvement (lacosamide, clonazepam, phenytoin, perampanel, topiramate, cannabidiol, ketogenic diet) or even worsened seizure activity (clobazam; Figure S3). Follow‐up EEG continued to show persistently disorganized activity, with asymptomatic seizures localized to the central right and midline regions (Figure 3D). His level of consciousness altered progressively. On last follow‐up at the age of 6.5 years, the patient demonstrated profound intellectual disability, limited autonomy in daily activities, and speech impairments, speaking only a few words.

At the age of 7 years, the patient experienced a 2‐month episode of refractory status epilepticus, with a suspected but unconfirmed diagnosis of limbic encephalitis. Management included admission to the intensive care unit and plasma exchange therapy. The myoclonic status epilepticus resolved following oral administration of phenytoin and zonisamide, with a gradual and complete return to the patient's baseline status: independent walking, presence of stereotypies, absence of spoken language, and emotional expression through smiling and vocalizations. No signs of pyramidal syndrome were observed.

### 3.3 Characterization of the structural rearrangements by LRS

Both patients had a de novo gain of minimal size of 536 kbp within the *FGF12* gene at the 3q28q29 locus as previously detected by array CGH analysis: arr[GRCh38] 3q28q29 (192 182 695_192 719 315) × 3. Targeted LRS was performed using the adaptive sampling method, focusing on the region chr3:163 000 000–195 000 000, which includes *FGF12* among more than 120 genes. After sequencing enrichment, the targeted region achieved 22‐fold coverage for both patients, whereas the entire genome had 2–3‐fold coverage. Sequencing generated 12.6 GB and 6.6 GB of data, corresponding to 14.9 million and 11.37 million reads for Patient 1 and Patient 2, respectively. The average supporting read size over the copy‐gain variant containing FGF12 was 15 508 bp for Patient 1 and 7948 bp for Patient 2.

**FIGURE 4:**
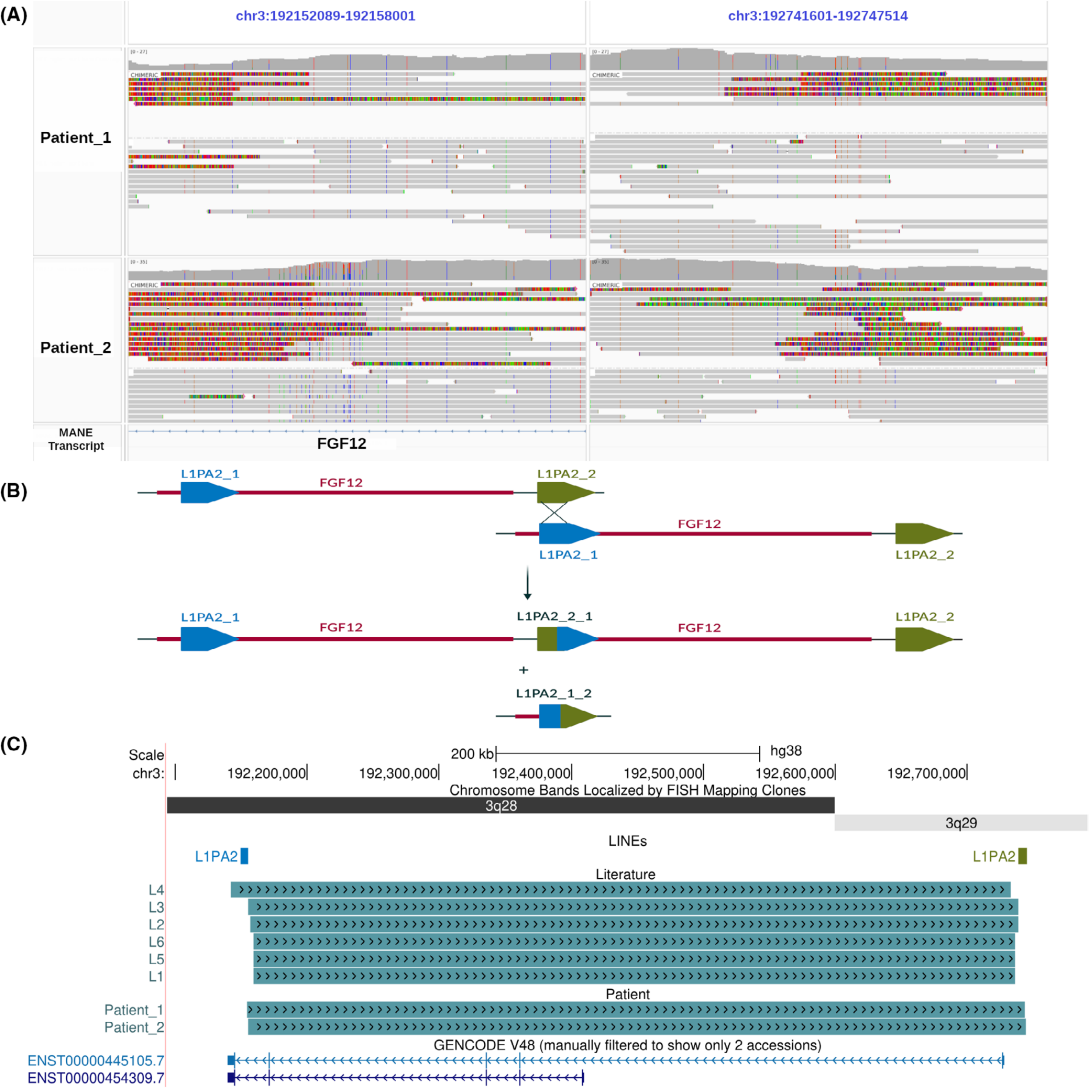
Study of DNA rearrangement encompassing *FGF12* gene. (A) Visualization of long‐read sequencing reads overlapping the FGF12 duplication breakpoints in Patients 1 and 2, displayed using the Integrated Genome Viewer. (B) Schematic representation of the repair mechanism through nonallelic homologous recombination, precisely localized on L1PA2 LINE elements (LINE‐1 Primate specific subfamily A, number 2 ; Long Interspersed Nuclear Element) at the *FGF12* duplication breakpoint. (C) Overview of duplications identified in patients from the literature (L1–L6) and in patients from our study (Patient 1 and Patient 2), along with the localization of L1PA2 elements in the UCSC Genome Browser (University of California, Santa Cruz; chr3:192 080 000–193 020 000). (FISH = Fluorescence In Situ Hybridization; MANE = Matched Annotation from NCBI and EMBL‐EBI).

The precise coordinates of the identified duplications were as follows: NC_000003.12:g.192153910_192743885dup in Patient 1 and NC_000003.12:g.192155125_192745081dup in Patient 2 (Figure 4A). Furthermore, Structural variant calling using CuteSV detected the duplication in both patients, further confirming these events. The breakpoints were located within the L1PA2‐A and L1PA2‐B (LINE‐1 Primate‐specific subfamily A, number 2) LINE elements (Long Interspersed Nuclear Element) at chr3:192 149 316–192 155 328 and chr3:192 739 214–192 745 242, respectively. These LINE elements share 98.14% sequence homology. Chimeric read alignments indicated a direct tandem duplication for both cases, with the 5′ breakpoint located in the last intron, common to both isoforms. The 3′ breakpoint was located downstream of the 5' untranslated region in an intergenic region (Figure 4B–4C). The involvement of L1PA2‐A and L1PA2‐B LINE elements in both patients suggests a nonallelic homologous recombination (NAHR) mechanism (Figure 4B). This mechanism and the occurrence of duplications are further supported by Sanger sequencing analysis of the breakpoints (Figure S4), which revealed homologous sequences of 73 bp in Patient 1 and 53 bp in Patient 2.

### 3.4 LRS of *FGF12* major isoforms A and B

cDNA analysis was performed on *FGF12* isoform A and isoform B. Both WT transcripts share the terminal exons, referred to as exons 3 to 6. They differ in their first exons; WT transcript A contains exon 1A, whereas WT transcript B contains exon 1B and exon 2. Exons 1A, 1B, and 2 together form the initial transcript exons (ITEs).

**FIGURE 5:**
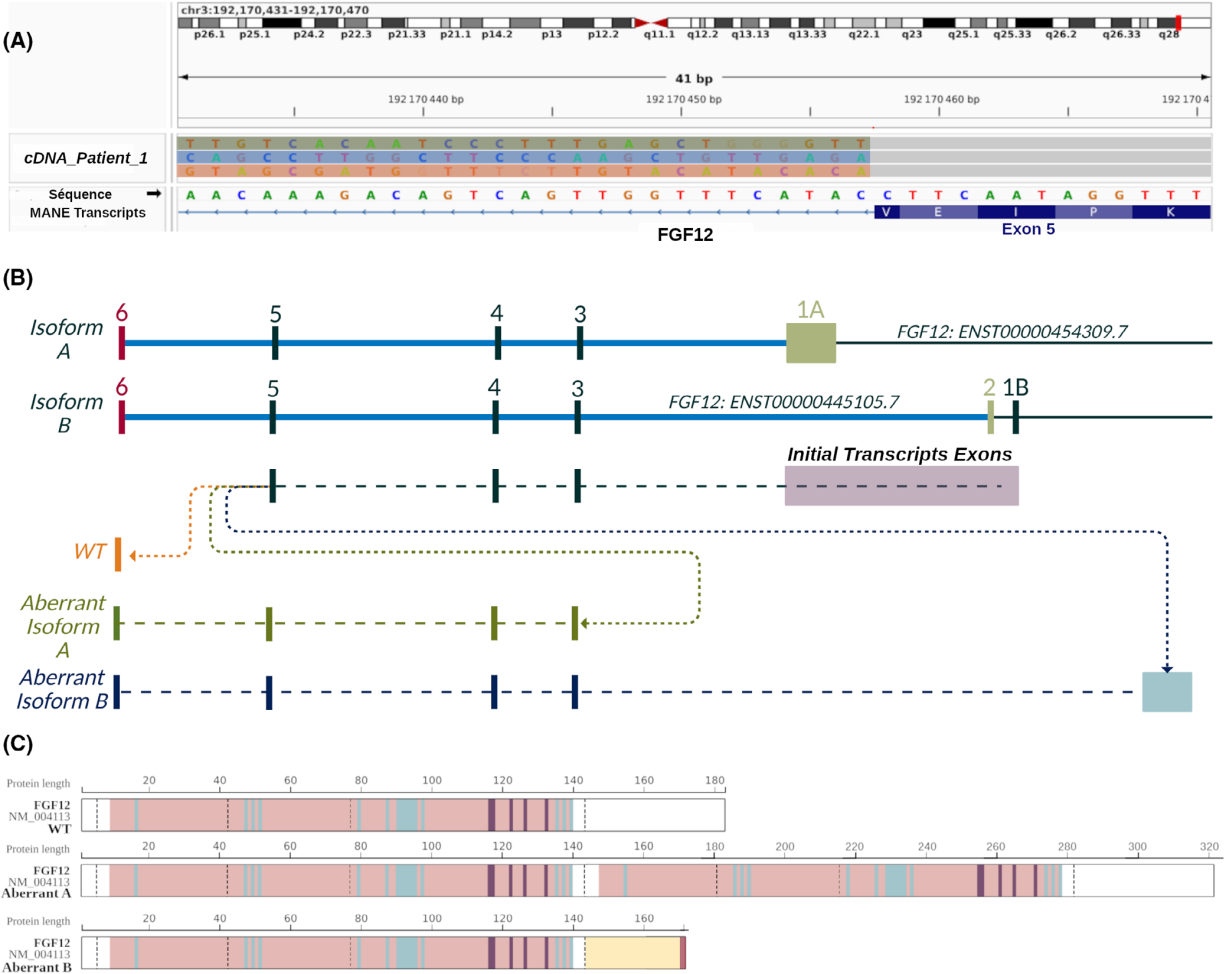
Study of the impact on *FGF12* RNA transcripts. (A) Visualization of long‐read aberrant transcripts from Patient 1 of exon 5, displayed using the Integrated Genome Viewer. (B) Modeling of the sequences of the wild‐type (WT) transcript isoforms (A and B) and aberrant transcripts based on the visualization of sequencing of transcripts. (C) Modeling of the sequence of the WT and aberrant proteins (yellow: intergenic sequence; red: stop codon). (MANE = Matched Annotation from NCBI and EMB‐EBI).

RNA was extracted from human LCLs derived from Patient 1 and reverse transcribed in cDNA fragments. PCR primers were designed to amplify potential aberrant transcripts, and the resulting amplicon library was sequenced using LRS technology. By analyzing the sequences of the last exons, we identified two different types of aberrant isoforms in addition to the WT isoforms (Figure 5A). The first, aberrant isoform A, involved a fusion of the coding sequences of exon 5 and exon 3, skipping exon 6, in the sequence ITE‐3‐4‐5‐3‐4‐5‐6 (Figure 5B). The second, aberrant isoform B, was characterized by the insertion of 123 nucleotides from the intergenic region chr3:192 738 861–192 738 983, named “INS,” after exon 5, followed by exon 3, in the sequence ITE‐3‐4‐5‐Ins‐3‐4‐5‐6 (Figure 5B). The WT proteins yielded from isoform A and isoform B consist of 243 and 181 amino acids (aa), respectively. The aberrant isoform A protein based on the isoform B is predicted to be 320 aa, with no premature termination due to the in‐frame fusion of exon 5 and exon 3 (Figure 5C). In contrast, the aberrant isoform B protein is predicted to be truncated with a premature stop codon (Figure 5C).

## 4 DISCUSSION

Resurgent sodium currents are essential for regulating the excitability of central and peripheral neurons. In this study, we applied LRS to investigate the structural organization of recurrent intragenic microduplications in *FGF12* and their effects on transcription in two patients with developmental and epileptic encephalopathy (DEE), both harboring an overlapping phenotype and specific EEG features.

Recurrent *FGF12* microduplications with breakpoints located within the L1PA2‐A and L1PA2‐B LINE elements have been previously identified in patients with DEE through array CGH and confirmed via Sanger sequencing (Table 1).^12^, ^13^, ^14^, ^21^ LRS enabled the precise identification of the complete structural rearrangement as a direct tandem duplication between the two interspersed paralogous repeats, L1PA2‐A and L1PA2‐B, as previously suspected.^12^, ^14^ Unlike whole‐genome short‐read sequencing, LRS provides an unbiased and comprehensive approach to investigate the two‐dimensional organization of the genome, thereby supporting the formulation of physiopathological hypotheses.^22^, ^23^ This structural change is likely to be driven by the NAHR mechanism, which exploits sequence homology between the directly oriented L1PA2‐A and L1PA2‐B repeats to mediate recombination. This process can result in either deletions or duplications, as observed in other genomic instability hotspots flanked by repeat elements.^24^ Similarly, the recurrent *FGF12* duplication exhibits highly conserved breakpoints, with homologous regions differing by only a few base pairs, further supporting an NAHR‐mediated mechanism.^12^, ^13^, ^14^, ^21^

**TABLE 1:** Clinical table of newly reported and previously published patients with the *FGF12* recurrent duplication of 500 kb and *FGF12* missense variant.

Characteristic	Patient 1	Patient 2	Shi et al. 2017	Verheyen et al. 2020	Abraham et al. 2024	Willemsen et al. 2020, Patient 1	Willemsen et al. 2020, Patient 2	Willemsen et al. 2020, Patient 3	Duplication synthesis	Missense variant synthesis, Trivisano et al., *n* = 16
Patient age at last consultation	6 years	6 years	8 years	7 years	22 years	10 years	5 years	30 years	6–22 years	1 month to 33 years
Genomic variant	3q28q29 (chr3:192 153 910–192 743 885) (hg38)	3q28q29 (chr3:192 154 622–192 744 509) (hg38)	3q28q29 (chr3:192 159 189–192 736 886) (hg38)	3q28q29 (chr3:192 156 263–192 739 147) (hg38)	3q28q29 (chr3:192 155 384–192 738 956) (hg38)	3q28q29 (chr3:192 142 300–192 733 325) (hg38)	3q28q29 (chr3:192 159 179–192 736 896) (hg38)	3q28q29 (chr3:192 159 179–192 736 896) (hg38)		
Inheritance	De novo		De novo	De novo	De novo	De novo	Inherited from Patient 3	Mother of Patient 2, de novo		
Age at diagnosis	2 years	5 months	NA	NA	16 years	NA	NA	NA		
Neurological impairment										
Intellectual impairment	Yes	Yes	Yes	Yes	Yes	Yes	Yes	Yes	Yes (100%)	Yes (87.5%)
Early psychomotor development	Normal		Normal	Normal	Normal		DD	DD	Normal (4/8), DD (2/8)	
Sitting age	NA	9 months	NA	NA	NA	NA	NA	NA		
Walking age	15 months	15 months	NA	NA	NA	NA	NA	NA		
Age of first words	12 months	2 words at 18 months (dad, mom)	NA	NA	NA	NA	NA	NA		
Current language level: oral [words, sentences], written [syllabic, fluent reading]	Absence of language	Few words	Few words	Single words	No speech (before her death at age 22 years)	No speech	Reduced vocabulary, drooling	Speech difficulties	Abnormal speech: 100%	
Autonomy in daily life: cleanliness, eating, dressing	Lack of autonomy	Lack of autonomy	Lack of autonomy, ataxic walking	Loss of practical hand skills, independent walking	Loss of the ability to speak and eat, wheelchair dependent	Wheelchair dependent, independently walking at 5 y.o.	Walking independently at 5 y.o	Care supervisor for her daily activities, limited writing and reading, special education	Reduced autonomy: 100%	
IQ	Profound ID	NA	Profound ID	NA	NA	Severe ID	NA	Mild to moderate	Mild to Profound	Mild to severe
ASD or autistic traits	Yes	Yes	NA	NA	NA	No	Yes, level 3	NA	Yes (23%), no (7%), NA (70%)	Yes (43.7%), no (25%), other (18.7%)
Neurological signs	Global hypotonia	NA	Aphasia, ataxia	Hand apraxia	NA	Ataxia	Uncoordinated gait	Unsteadiness		
Behavioral issues	Poor eye contact	Short attention span	NA	Inappropriate laughing and crying	NA	NA	NA	NA		
Sleep disturbance	Yes	No	Yes	Yes	NA	NA	NA	NA		
Long‐term treatment	DCI, BZD, PB, CZP	2 types of LTG	PHT	LTG, LEV, TPM, PHT, BZD	PHT, VPA, GBP	VPA, BZD, LTG, TPM	VPA	VPA, CBZ	DCI (1/8), BZD (3/8), PB (1/8), CZP (1/8), LTG (4/8), PHT (2/8), LEV (1/8), TPM (2/8), VPA (4/8), CBZ (1/8), GBP (1/8)	
Epilepsy										
Febrile seizure: yes/no	No	No	No	No	No	No	Yes, 13 months	No	No (7/8)	No (100%)
Febrile seizures, *n*	0	0	0	0	0	0	0	0		
Febrile seizure: start/end age	NA	NA	NA	NA	NA	NA	NA	NA	NA	NA
Age at onset	18 months	5 months	3 years	4 y.o	22 months	12 months	15 months	1 month	19 months, range = 1 month to 4 years	24 days, range = 2 days to 4 months
Seizure type	Recurrent nonfebrile status epilepticus	Absence, then clonic	Tonic–clonic, brief tonic or complex partial seizures	Generalized, tonic, and tonic–clonic	Provoked generalized convulsion	Tonic–clonic, atonic, tonic, myoclonic, autonomic	Cluster of generalized tonic–clonic	Generalized tonic–clonic		TS (7/16), FTS (8/16), FBTCS (8/16), MS (2/16), FS (5/16), AS (1/16), A (1/16), ES (2/16)
Status epilepticus	Yes		NA	Yes	Yes				Yes (3/8)	
Generalized epilepsy: yes/no	Yes	Yes	Yes	Yes	Yes	Yes	Yes	Yes	Yes (100%)	Yes (44%)
Partial epilepsy: yes/no	Yes	Yes (temporo‐occipital, left)	Yes	NA	No	No	No	No	Yes (38%)	Yes (87%)
Drug resistance	Yes	Yes	Yes	Yes	No	Yes	No	Yes	Yes (75%)	Yes (44%)
Current treatment	CZP, DCI, PB, BZD	LTG 600 mg 2/day, TPM 2/day, CZP	PHT	LTG, LEV, TPM, CLB	No	VPA, BZD, LTG, TPM	VPA	VPA, CBZ		
Frequency of seizures	Multiday	1 per month	Daily seizure at start	NA	Regular	Periods of up to 6–8 weeks with very few seizures, often followed by several weeks of daily seizures	NA	Regular breakthrough seizures	Frequent 50%	Frequent (31%), infrequent (6%), no (31%), NA (13%), twice (13%), monthly (6%)
Treatments already used	VPA, LTG, CZP	LCM, BZD	PB, BZD, VPA, Kbr	VPA, PHT, CBZ, plasmapheresis	PHT, VPA, GBP	NA	VPA	NA		PB (13/16), VPA (8/16), PHT (11/16), GVG (5/16), TPM (6/16), CZP (1/16), PN (3/16), LEV (9/16), KD (3/16), PER (3/16), VNS (3/16), PRG (1/16), CBZ (7/16), LTG (2/16), RUF (1/16), CLB (2/16), ESM (1/16), ZNS (1/16), Kbr (1/16), RTG (1/16), OXC (1/16), LCM (1/16), CLZ (1/16), steroids (1/16), PLP (2/16), folic acid (1/16), biotin (1/16)
Effective treatment			PHT	PHT, LTG, LEV, TPM, CLB	PHT, VPA, GBP	VPA, BZD, LTG, TPM	VPA	VPA, CBZ	PHT (3/8), VPA (4/8), LTG (2/8), LEV (1/8), BZD (2/8), TPM (2/8), CBZ (1/8), GBP (1/8)	PHT (8/16), VNS (1/16), CBZ (2/16), RUF (1/16), LTG (1/16), PB (2/13), VPA (2/16), ASM (2/16)
Aggravating treatment	No	NA	NA	NA	NA	NA	No	NA	Not reported	
Regression of acquisitions	Yes at 20 months	Yes	Yes	Yes	Yes, 7 y.o	Yes, 3 y.o	No	No	Yes (6/8 75%)	
EEG, first seizure background trace	Disorganized background activity	Diffuse delta hypersynchrony of sleep onset, ample, then polymorphic delta rhythm overloaded by sleep spindles, slow Rolandic spikes Wakefulness: low‐voltage theta rhythm, symmetrical	NA	NA	NA	NA	At 13 months: normal EEG	NA		Interictal: slow (56%), normal (31%), no description (23%)
EEG, first seizure paroxysmal abnormalities	Rare focal spikes of variable focalization, often diffuse over one hemisphere	Left temporo‐occipital paroxysmal wave spikes	NA	NA	NA	NA	At 13 months: normal EEG	NA		Interictal: multifocal SW (62%), diffuse SW (6%), focal SW (6%), suppression burst (6%)
EEG, follow‐up background trace	Background of diffuse encephalopathy rich in bi‐ and triphasic delta activity		Severe suppression of background activity	Background suppression when NCSE; or sleep background, diffuse slowing	At 8 y.o: normal EEG	At 3 y.o: slowed background activity	NA	NA	Abnormal background (4/5)	Slow (64%), normal (14%), no description (22%)
EEG, follow‐up paroxysmal anomalies	Isolated generalized spikes or bursts at 2.5 Hz, with atypical absence and concomitant myoclonus		Paroxysmal bursts	Periodic bursts of slow wave activity (NCSE); or primary generalized and bioccipital epileptic discharges	At 8 y.o: normal EEG	At 3 y.o: multifocal epileptic discharges	At 2 y.o: generalized epileptic activity	NA	Paroxysmal abnormality (5/6)	Multifocal SW (64%), focal SW (21%), generalized and focal paroxysmal in temporal regions (15%)
MRI										
MRI abnormalities: description and picture if possible	At 4 y.o: corticosubcortical atrophy	2018: normal MRI 2024: nodular lesion of the same signal as the 5 mm cortex of the white matter of the left cerebellar peduncle; probable gray matter heterotopia	At 8 y.o: mild cerebral and cerebellar atrophy	NA	At 18 y.o: T2 hyperintensity in left posterior periventricular white matter consistent with a perivascular space	Bilateral delayed myelination in the parieto‐occipital region	Mild prominence of the subarachnoid space in the frontal regions bilaterally, otherwise normal	Normal		Cerebellar atrophy (19%), normal (38%), mild cerebral atrophy (6%), temporal sclerosis/cerebellar folia (6%), cerebral atrophy (19%), mild Chiari (6%), mild enlargement of lateral ventricle (6%)
Pyramidal signs, spasticity, hyperreflexia	Progressive spasticity of the 4 limbs	No	No	NA	NA	NA	NA	NA		
Other										
Height, SD	At 6 y.o: −1 SD	−0.5 SD	At birth: −1.2 SD	NA	NA	NA	NA	NA		
Weight, SD	At 6 y.o: M	−0.5 SD	At birth: −0.5 SD	NA	NA	NA	NA	NA		
Head circumference, SD	At 6 y.o: −1 SD	NA	At birth: −0.4 ET	NA	NA	NA	NA	NA		
Appetite disorders: food selection, small appetite	No	No	Dysphagia when seizures occurred	NA	NA	Progressive feeding difficulties, reflux	NA	NA		
Enteral nutrition, gastrostomy	Gastrostomy for nutritional indications (December 2020)	No	Gastrostomy	Gastrotomy at one moment	Gastrostomy	Tube feeding (PEG)	NA	NA		
Refraction disorders	Cortical blindness	No	NA	NA	NA	NA	NA	NA		
Deafness	No	Hypoacusis, transtympanic aerators, then normal hearing	NA	NA	NA	NA	NA	NA		
Other	Constipation	NA	Dyspnea	No	No	Upper airway infection, Tachycardia during prephase	Dysmorphic features	No		

Abbreviations: A, Absence seizures; AS, Atypical absence seizures; ASD, autism spectrum disorder; ASM, Anti seizure medication; BZD, benzodiazepines; CBZ, carbamazepine; CLB, clobazam; CLZ, clorazepate; CZP, clonazepam; DD, developmental delay; DCI, acetazolamide; EEG, electroencephalography; ES, Epileptic spasms; ESM, ethosuximide; ET, Essential tremor; FBTCS, focal to bilateral tonic–clonic seizures; FS, Febrile seizure; FTC, Focal to tonic seizures; GBP, gabapentin; GVG, vigabatrin; ID, intellectual disability; IQ, intelligence quotient; Kbr, Potassium bromid; KD, ketogenic diet; LCM, lacosamide; LEV, levetiracetam; LTG, lamotrigine; M, median; MRI, magnetic resonance imaging; MS, Myoclonic seizures; NA, information not available; NCSE, nonconvulsant status epilepticus; OXC, oxcarbazepine; PB, phenobarbital; PEG, percutaneous endoscopic gastrostomy; PER, perampanel; PHT, phenytoin; PLP, pyridoxal phosphate; PN, prednisolone; PRG, pregabalin; RTG, retigabine; RUF, rufinamide; SW, Slow waves; TPM, topiramate; TS, Tonic seizures; VNS, vagus nerve stimulation; VPA, valproic acid; y.o, years old; ZNS, zonisamide.

As member of the FHF subfamily, *FGF12* has at least two transcription initiation sites that generate two major isoforms. Transcript A (ENST00000454309.7; NM_021032) encodes FGF12 isoform A, a protein with a longer N‐terminal sequence compared to FGF12 isoform B, encoded by transcript B (ENST0000044105.7; NM_004113). Structurally, FHF proteins share a β‐trefoil fold, with a conserved C‐terminal region that covers a hydrophobic surface on the β‐trefoil fold.^2^, ^3^, ^25^, ^26^ The β‐trefoil fold of FGF12 contains unique motifs that facilitate interactions with the cytoplasmic tails of Nav channels, thereby modulating their activity. These interactions enhance neuronal excitability by shifting the voltage dependence of rapid inactivation of the Nav channels.^2^, ^3^, ^26^ FGF12 contains key surface residues essential for Nav channel interactions. The FGF12 isoform A possesses distinct properties due to a conserved motif of 15–20 residues at the N‐terminus, which acts as an open‐channel blocking particle. This motif facilitates both rapid and prolonged inactivation by maintaining the channel in a long‐term inactivated state.^2^, ^26^ A pathogenic missense variant, p.(Arg144His) in isoform A, has been identified as a cause of DEE. This substitution disrupts the interaction FGF12–Nav channel interaction, causing a depolarizing shift in channel inactivation and leading to a gain‐of‐function effect.^3^, ^5^, ^15^

The recurrent *FGF12* duplications identified in this study included the central motif and key residues essential for interactions with Nav channels, specifically Lys9, Ile11, Arg52, Tyr93, and Val95 (as designated in FGF12 isoform A nomenclature).^15^ These duplications were associated with two aberrant transcripts, each originating from transcription starts of either transcript A or transcript B. The first aberrant transcript, derived from WT transcript A, is composed of eight exons, corresponding to the following sequence: exon 1A‐3‐4‐5‐3‐4‐5‐6, with no predicted occurrence of a premature stop codon. The second aberrant transcript, derived from WT transcript B, is composed of seven exons and includes an intergenic region. It corresponds to the following sequence: exon 1B‐2‐3‐4‐5‐INSsequence‐3‐4‐5‐6 (Figure 5). Previous studies using RT‐PCR and Sanger sequencing on blood and hair follicles from a patient with *FGF12* duplication suggested the presence of a long aberrant transcript containing all exons in‐frame. This hypothesis is further supported by our study, with the identification by LRS on cDNA of the presence of the same extended transcript, corresponding to aberrant transcript A.^14^, ^15^ The aberrant transcript could potentially be fully translated, yielding a hypothetical longer protein with two central β‐trefoil folded regions, as exons 5 and 3 remain in‐frame, preventing a frameshift. Although we have not analyzed the protein sequence, we suspect that the aberrant structural conformation may lead to protein instability and degradation. Our results also validated the existence of a second aberrant transcript characterized by an out‐of‐frame insertion of intergenic content, leading to a premature stop codon.^14^ It is also important to note that *FGF12* is affected by both recurrent and nonrecurrent structural variants. For example, a patient with biallelic structural variants—including a deletion of exon 6 and a duplication of exons 3–4 of *FGF12*—was recently reported.^27^ In that study, LRS enabled the resolution of small, complex biallelic structural variants. The patient exhibited focal motor seizures progressing to bilateral tonic–clonic seizures and status epilepticus, with apparent treatment responsiveness. These findings illustrate the heterogeneity of *FGF12*‐related rearrangements and emphasize the utility of LRS in characterizing clinically relevant, complex structural variants.

Pathogenic missense variants have been linked to a gain‐of‐function effect on Nav channels, whereas duplications have been linked to loss of function. Despite these opposite mechanisms, both alterations give rise to an identical DEE phenotype, differing only by the age at epilepsy onset. Gain‐of‐function variants lower the firing threshold of Nav channels, leading to neuronal hyperexcitability and early onset seizures.^5^, ^15^ In contrast, loss‐of‐function duplications do not trigger hyperexcitability and are associated with a slightly later seizure onset. The putative aberrant elongated variant protein may shift the voltage dependence of Nav channel inactivation toward more hyperpolarized potentials, impairing channel activity, further supporting a loss‐of‐function mechanism.^15^ In the hippocampus, mossy fibers carry signals from granule cells in the dentate gyrus to the CA3 region. These fibers target excitatory pyramidal cells and GABAergic inhibitory interneurons. These interneurons, which release the neurotransmitter GABA, play a key role in suppressing neuronal activity, thereby regulating excitation and maintaining network balance.^28^, ^29^, ^30^ Nav channels, particularly Nav1.2, are highly expressed in the dentate gyrus granule cells, which project to the CA3 region. Interestingly, mossy fibers form connections with specific inhibitory interneurons known as basket cells approximately 50 times more frequently than with excitatory pyramidal cells. Loss of function in these Nav channels reduces the efficiency of inhibitory signaling, thereby increasing hyperexcitability in the CA3 region.^28^, ^29^, ^30^ This altered excitability may explain the later onset of epileptic seizures (average onset at 19 months for duplicated variant against 19 days for missense variants), a timing that corresponds with the expression of these cells in a more mature brain and is observed in patients with *FGF12* duplication.^5^, ^12^, ^13^, ^14^, ^15^ Thus, as previously suspected, patients with *FGF12* duplication presented with a later onset of seizures and showed an apparent stagnation or regression in development after seizure onset.^12^

**FIGURE 6:**
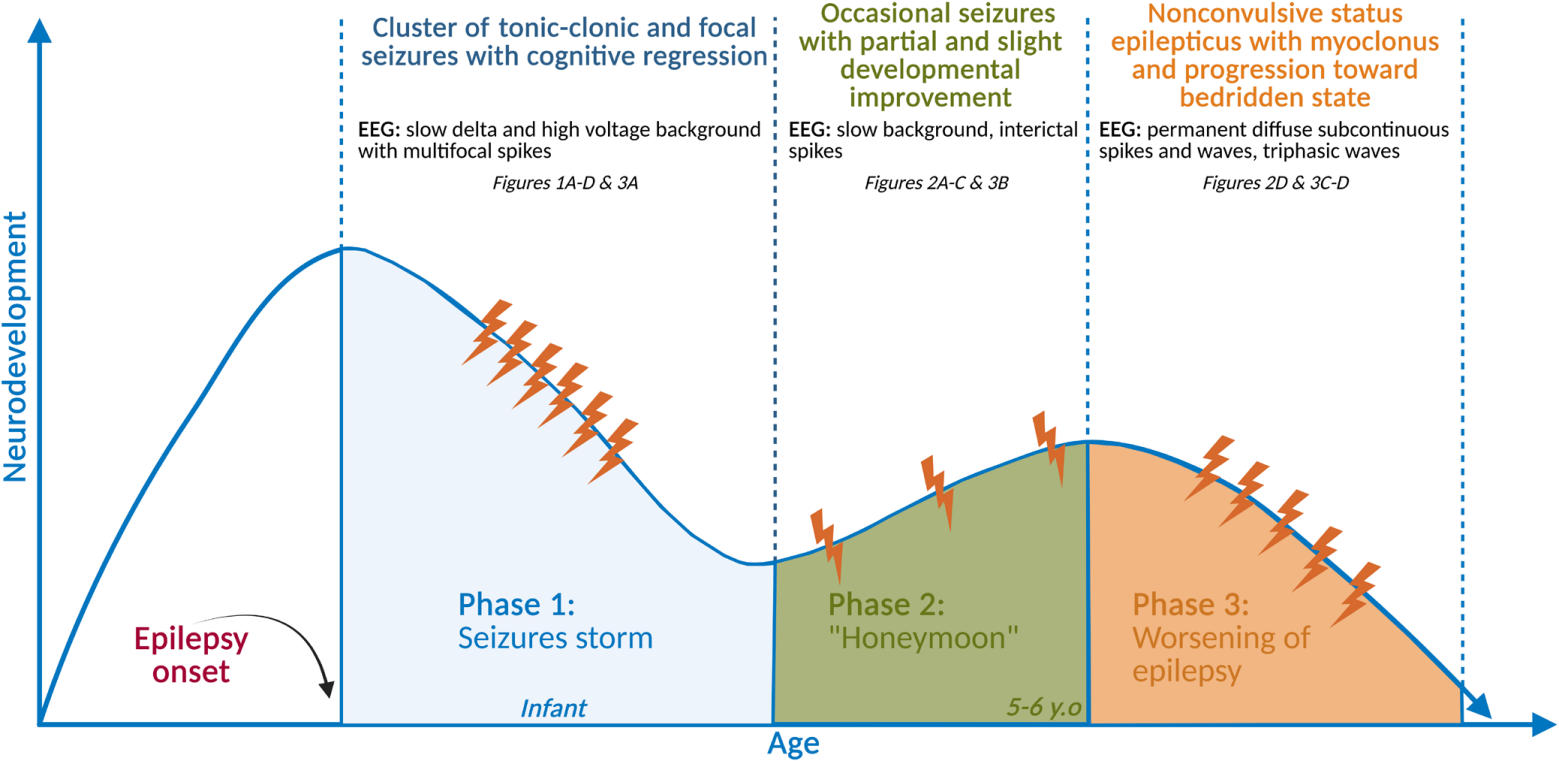
Three‐phase evolution of epilepsy and neurodevelopment. Following initial development, patients experience a seizure storm (phase 1), a transient improvement (phase 2), and a later decline with nonconvulsive status epilepticus and motor deterioration (phase 3). EEG, electroencephalography; y.o, years old.

Most of patients with *FGF12* pathogenic missense variants presented with drug‐resistant epilepsy and EEG showing mainly multifocal nonspecific abnormalities. When EEG data were available, half presented with slow background activity and interictal focal or multifocal spikes; discontinuous patterns of suppression burst were rarely seen. Ictal EEG was reported as focal (with rapid spread to bilateral regions) or generalized with low‐voltage fast activity, followed by widespread spikes and postictal suppression.^9^, ^10^ Conversely, in patients with *FGF12* duplications, we have observed a more complex EEG pattern, with some common specificities (Table 1). First, at the onset of epilepsy, both patients presented a marked alteration in electrogenesis compatible with severe encephalopathy, suggesting an inflammatory etiology. Both patients showed a very slow and high‐voltage activity, as described in encephalitis.^31^ One of our patients, like others in the literature, was treated with immunomodulatory drugs.^21^ In treated patients, a three‐phase evolution was observed. The first phase corresponded to a storm of seizures with cognitive regression (Figure 6). Phase 2 was a “honeymoon phase,” with partial response to therapy, corresponding to a seizure‐free period, but with significant improvement in cognitive performance. Finally, in phase 3, we observed a worsening of the epilepsy with nonconvulsive status epilepticus, obnubilation, myoclonus (accompanied by diffuse subcontinuous spikes and waves with abundant triphasic waves on EEG), and progression toward bedridden state.^12^, ^14^, ^21^ Both patients reported in this study had bilateral tonic–clonic seizures and focal temporal seizures at epilepsy onset. This temporal localization is consistent with the hypothesis of disruption in the balance between excitatory and inhibitory signaling within these regions.^28^ The kinetics of seizure onset and progression in *FGF12* duplications may suggest a disease course similar to Nav1.2 (SCN2A) loss of function, with a later onset compared to Nav1.2 (SCN2A) gain of function.^28^ The parallel with Nav1.2 alterations could be extended to treatment approaches. Like patients with pathogenic missense variants, those with duplications exhibited significant resistance to antiepileptic treatments despite various therapeutic approaches. Although phenytoin demonstrated partial efficacy in *FGF12* duplications, its overall impact was limited and failed to produce substantial clinical improvement.^10^, ^12^, ^14^

We have provided the first comprehensive characterization of *FGF12* duplications using LRS approaches on DNA combined with cDNA analysis, to reveal the complete structural rearrangement caused by NAHR mechanisms and their impact on transcripts. This resulted in two distinct aberrant isoforms: an elongated isoform A and a frameshift isoform B. The electroclinical consequences of *FGF12* duplications appear to be more specific than previously suspected compared to point pathogenic missense variants, with clinical regression observed after seizure onset and distinctive EEG patterns at onset and during evolution/progression, suggesting that these duplications could be suspected based on EEG findings.

## AUTHOR CONTRIBUTIONS

Jade Fauqueux and Thomas Smol wrote the manuscript. Jade Fauqueux and Caroline Thuillier conducted molecular genetic studies, assisted by Céline Villenet. Jean‐Pascal Meneboo, Emilie Ait‐Yahya, and Martin Figeac carried out the bioinformatic analysis for long‐read genome sequencing. Jade Fauqueux handled the bioinformatics analysis for long‐read amplicon sequencing. Jade Fauqueux and Thomas Smol interpreted the LRS data. Elise Boudry and Nicolas Gruchy performed the array CGH analysis. Roseline Caumes, Laurence Chaton, Pierre Cleuziou, Adeline Trauffler, Sylvie Nguyen‐The‐Tich, Nicolas Gruchy, Marion Gerard, Simon Boussion, Nathalie Bach, and Anne‐Sophie Diependaële collected and evaluated clinical and genetic data. Thomas Smol and Jamal Ghoumid revised the manuscript. All authors contributed to discussions of the results, provided comments on the manuscript, and approved the final version.

## FUNDING INFORMATION

This work was supported by the University Hospital of Lille, France (Budget Projet Innovation).

## CONFLICT OF INTEREST STATEMENT

None of the authors has any conflict of interest to disclose.

## ETHICS STATEMENT

We confirm that we have read the Journal's position on issues involved in ethical publication and affirm that this report is consistent with those guidelines. The authors assert that all procedures contributing to this work comply with the ethical standards of the relevant national and institutional committees on human experimentation and with the Helsinki Declaration of 1975, as revised in 2013. This study was approved by the Comité de Protection des Personnes ethics committee (reference #2023‐A00473‐42).

## PATIENT CONSENT STATEMENT

All study participants signed written informed consent prior to their inclusion in the study.

## Supporting information


**Figure S1.** Primer positions for *FGF12* transcript amplification. Green arrows indicate primers spanning exons 3/4 to 6, which amplify both wild‐type and aberrant transcripts. Red arrows indicate primers spanning exons 4 to 3 (reverse orientation), specifically amplifying aberrant transcripts only.

**Figure S2.** Brain magnetic resonance imaging of Patient 1 at age 4 years. (A, B) Axial T1‐weighted images. (C, D) Sagittal T1‐weighted images. (E) Fluid‐attenuated inversion recovery image.

**Figure S3.** Chronological timeline of therapeutic treatment. (A) Chronological timeline of therapeutic treatments for Patient 1. The red dashed lines indicate the duration of each treatment. (B) Chronological timeline of therapeutic treatments for Patient 2.

**Figure S4.** Sanger sequencing of the tandem duplication breakpoints. (A) Sanger sequencing of the tandem duplication breakpoint in Patient 1, showing a 73‐bp homologous sequence. (B) Sanger sequencing of the tandem duplication breakpoint in Patient 2, showing a 53‐bp homologous sequence. Color legend: Green indicates the end of the duplication, blue indicates the beginning of the duplication, purple represents the common sequence of the two L1PA2 elements (LINE‐1 Primate‐specific subfamily A, number 2), and red denotes the nonhomologous sequence between the two L1PA2 elements.

## Data Availability

The data that support the findings of this study are available from the corresponding author upon reasonable request.
