# Supplementary figures and images for: Long‐read sequencing of recurrent FGF12 duplications in epilepsy: Insights into structural mechanisms and aberrant isoforms
Source: Epilepsia. 2025 Aug 21;66(12):5014–32. doi: 10.1111/epi.18609 (PMC12779332; doi:10.1111/epi.18609)

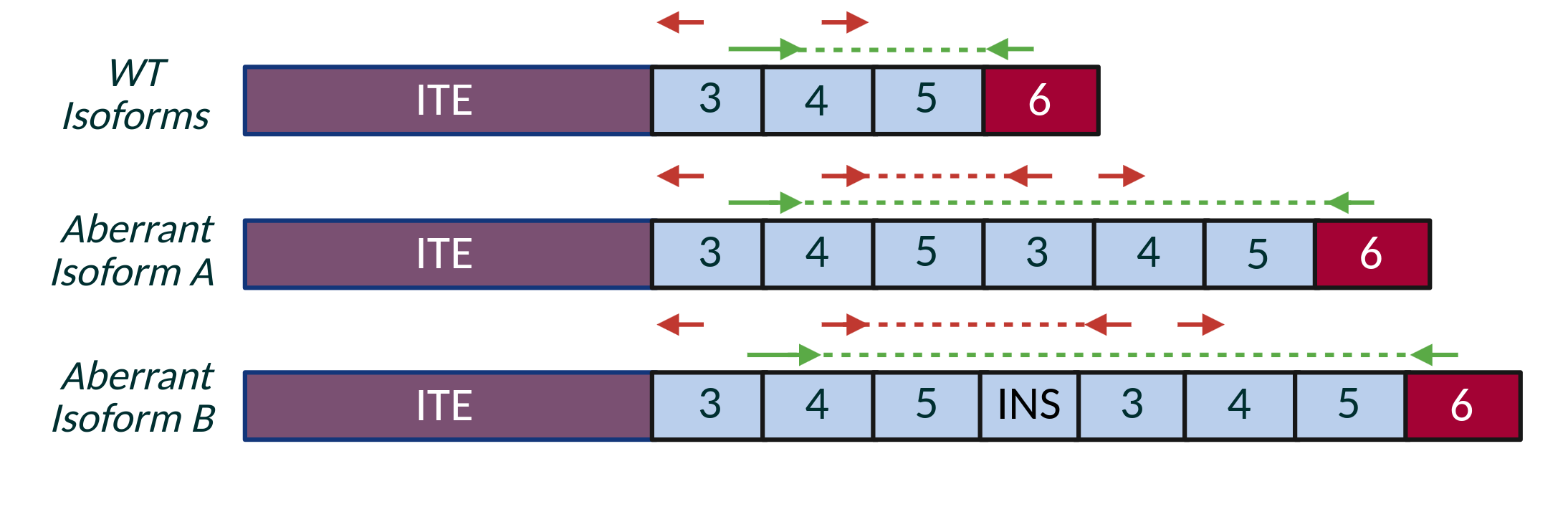

Supplement: Supplementary file 1 — Figure S1. Primer positions for FGF12 transcript amplification. Green arrows indicate primers spanning exons 3/4 to 6, which amplify both wild‐type and aberrant transcripts. Red arrows indicate primers spanning exons 4 to 3 (reverse orientation), specifically amplifying aberrant transcripts only. [file EPI-66-5014-s004.tif]

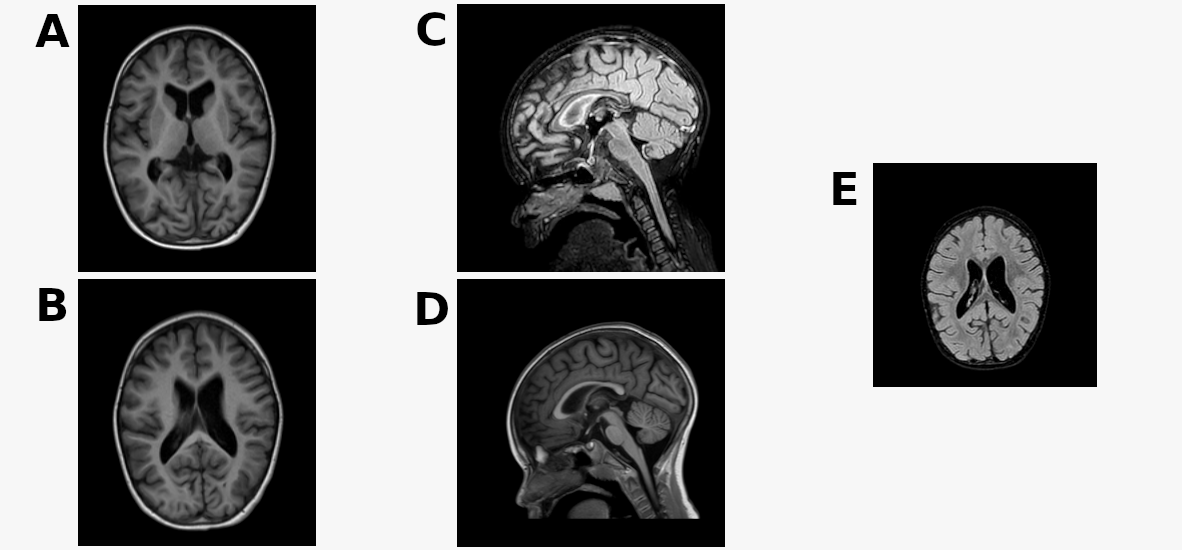

Supplement: Supplementary file 2 — Figure S2. Brain magnetic resonance imaging of Patient 1 at age 4 years. (A, B) Axial T1‐weighted images. (C, D) Sagittal T1‐weighted images. (E) Fluid‐attenuated inversion recovery image. [file EPI-66-5014-s001.tif]

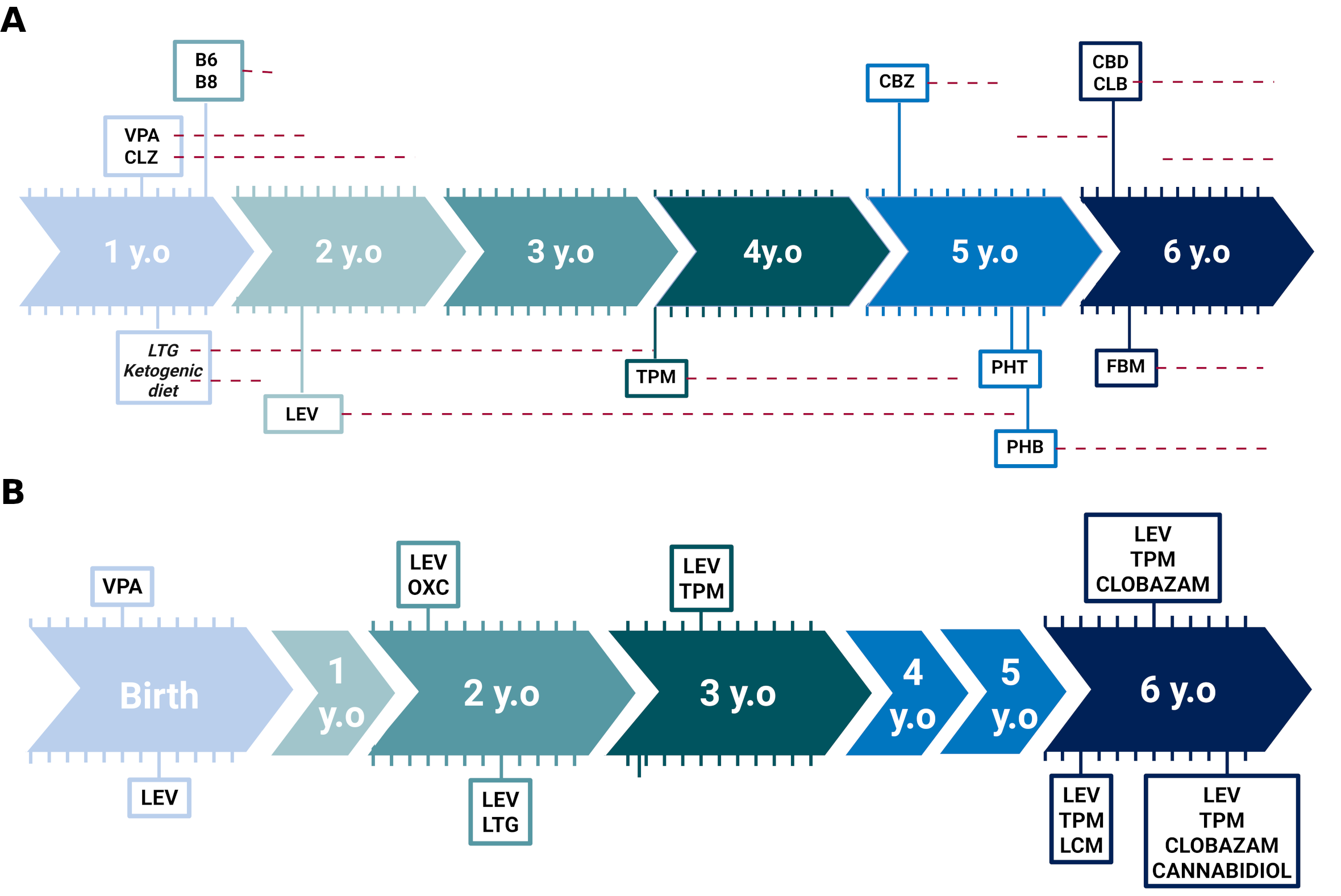

Supplement: Supplementary file 3 — Figure S3. Chronological timeline of therapeutic treatment. (A) Chronological timeline of therapeutic treatments for Patient 1. The red dashed lines indicate the duration of each treatment. (B) Chronological timeline of therapeutic treatments for Patient 2. [file EPI-66-5014-s002.tif]

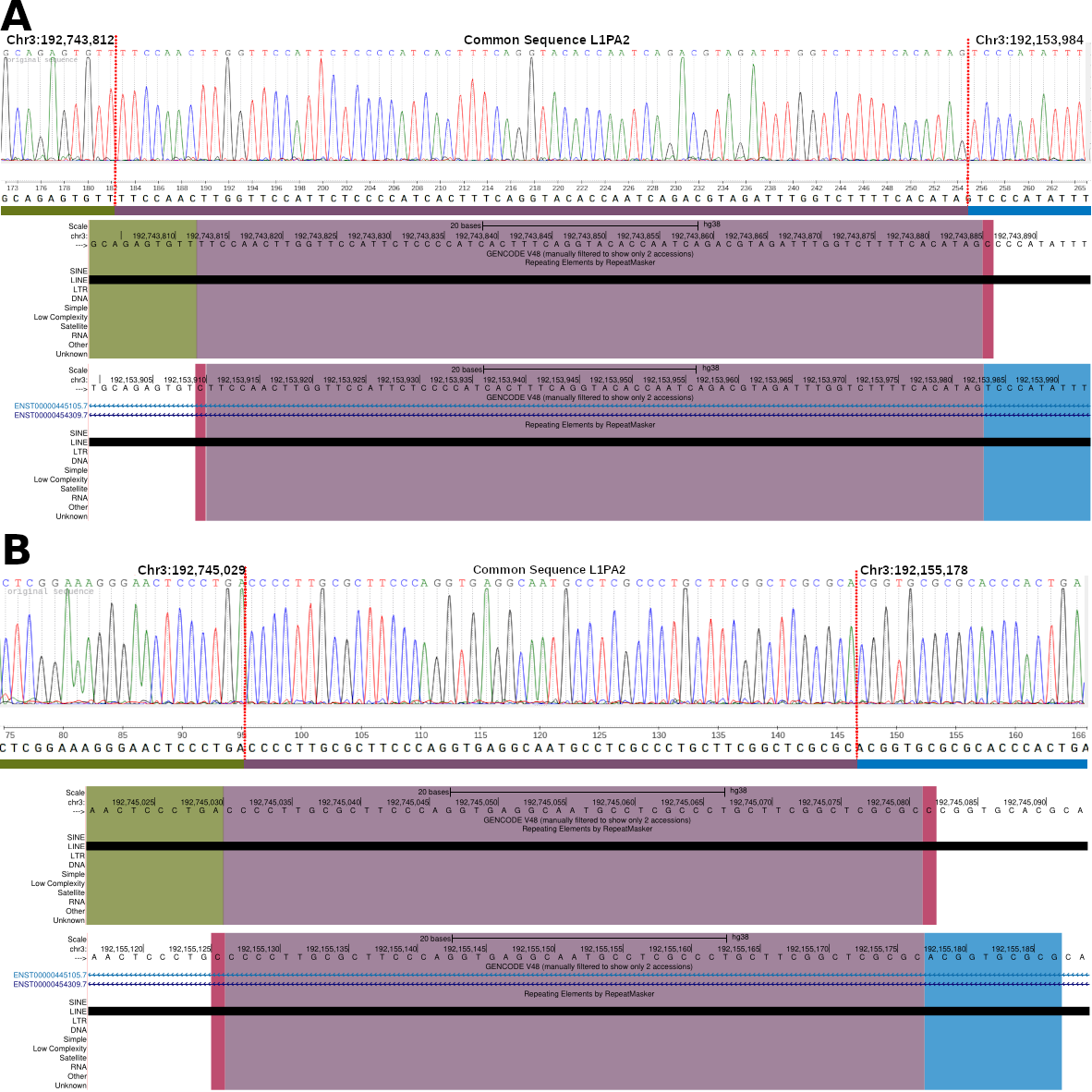

Supplement: Supplementary file 4 — Figure S4. Sanger sequencing of the tandem duplication breakpoints. (A) Sanger sequencing of the tandem duplication breakpoint in Patient 1, showing a 73‐bp homologous sequence. (B) Sanger sequencing of the tandem duplication breakpoint in Patient 2, showing a 53‐bp homologous sequence. Color legend: Green indicates the end of the duplication, blue indicates the beginning of the duplication, purple represents the common sequence of the two L1PA2 elements (LINE‐1 Primate‐specific subfamily A, number 2), and red denotes the nonhomologous sequence between the two L1PA2 elements. [file EPI-66-5014-s003.tif]
